# Amplifying post-stimulation oscillatory dynamics by engaging synaptic plasticity with transcranial alternating current stimulation

**DOI:** 10.3389/fnetp.2025.1621283

**Published:** 2025-07-18

**Authors:** Jeremie Lefebvre, Aref Pariz

**Affiliations:** ^1^ Department of Biology, University of Ottawa, Ottawa, ON, Canada; ^2^ Department of Physics, University of Ottawa, Ottawa, ON, Canada; ^3^ Krembil Brain Institute, University Health Network, Toronto, ON, Canada; ^4^ Department of Mathematics, University of Toronto, Toronto, ON, Canada; ^5^ Institute of Mental Health Research at The Royal, Ottawa, ON, Canada

**Keywords:** brain stimulation, post-stimulation after-effects, stimulation-induced, heterogeneity, neurons timescale diversity, network physiology

## Abstract

**Introduction:**

Periodic brain stimulation (PBS) techniques, either intracranial or non-invasive, electrical or magnetic, represent promising neuromodulatory tools for the treatment of neurological and neuropsychiatric disorders. Through the modulation of endogenous oscillations, PBS may engage synaptic plasticity, hopefully leading to persistent lasting effects. However, stabilizing such effects represents an important challenge: the interaction between induced electromagnetic fields and neural circuits may yield highly variable responses due to heterogeneous neuronal and synaptic biophysical properties, limiting PBS clinical potential.

**Methods:**

In this study, we explored the conditions on which transcranial alternating current stimulation (tACS) as a common type of non-invasive PBS leads to amplified post-stimulation oscillatory power, persisting once stimulation has been turned off. We specifically examined the effects of heterogeneity in neuron time scales on post-stimulation dynamics in a population of balanced Leaky-Integrate and Fire (LIF) neurons that exhibit synchronous-irregular spiking activity.

**Results:**

Our analysis reveals that such heterogeneity enables tACS to engage synaptic plasticity, amplifying post-stimulation power. Our results show that such post-stimulation aftereffects result from selective frequency- and cell-type-specific synaptic modifications. We evaluated the relative importance of stimulation-induced plasticity amongst and between excitatory and inhibitory populations.

**Discussion:**

Our results indicate that heterogeneity in neurons’ time scales and synaptic plasticity are both essential for stimulation to support post-stimulation aftereffects, notably to amplify the power of endogenous rhythms.

## Introduction

Brain stimulation has attracted significant interest in the last decades (Takeuchi and Berényi, 2020; Gschwind and Seeck, 2016; Bronstein et al., 2011). Various simulation techniques have shown promising results, and more are coming. Researchers, experimentally and theoretically, have addressed numerous challenges related to the effects of these interventions on behaviour (Miniussi and Vallar, 2011; Bestmann et al., 2015), brain function (Polanía et al., 2018), as well as pathologies such as epilepsy (Takeuchi and Berényi, 2020; San-Juan et al., 2022), Parkinson’s (Benninger et al., 2010; Madadi Asl et al., 2023), major depressive disorder (MDD) (Riddle et al., 2020; Haller et al., 2020) and stroke (Schlaug et al., 2008; Monti et al., 2013). Despite these promising results, it is still unclear how brain stimulation interventions shape endogenous brain dynamics (Ali et al., 2013; Helfrich et al., 2014; Alagapan et al., 2016; Reato et al., 2010) and the neural circuits that support them (Zaehle et al., 2010; Pariz et al., 2023). Indeed, brain stimulation outcomes remain variable: induced changes in neuron excitability vary remarkably between stimulation sites, repeated trials, and subjects, oftentimes vanishing after stimulation offset (Vogeti et al., 2022; Maeda et al., 2000; Eldaief et al., 2011; López-Alonso et al., 2014; Temperli et al., 2003). Uncovering the source of this variability can help to optimize existing brain stimulation paradigms and stabilize their effect on brain dynamics and plasticity.

Periodic brain stimulation (PBS) techniques, such as transcranial alternating current stimulation (tACS), repetitive transcranial magnetic stimulation (rTMS), and deep brain stimulation (DBS) have repeatedly been shown to be capable of altering neurons’ dynamics to interfere with cortical rhythms (Helfrich et al., 2014; Alagapan et al., 2016; Kasten et al., 2022; Kasten et al., 2016; Vossen et al., 2015; Negahbani et al., 2018; Herrmann et al., 2016; Krause et al., 2019; Nowotny et al., 2003; Lubenov and Siapas, 2008), thereby engaging synaptic plasticity by altering the neurons’ dynamics, firing rates and spike-timing (Sjöström et al., 2001) by modulating phase- and/or mode-locking beahviour of neurons (Pariz et al., 2023; Farokhniaee and Large, 2017) to alter network connectivity (Madadi Asl et al., 2023; Kromer and Tass, 2022; Kromer and Tass, 2024). However, the effects of these various types of stimulation may generate widely variable responses, notably due to physiological differences among neurons, while engaging different forms of brain plasticity (Shen et al., 2003). In fact, neural plasticity has been shown to depend on the stimulation frequency (Lea-Carnall et al., 2017; Yamawaki et al., 2012), highlighting the importance of tuning stimulation parameters to elevate its effects.

tACS is thought to work by engaging endogenous oscillations through time-varying electromagnetic waveforms and altering mode-locking behavior via continuous currents (Farokhniaee and Large, 2017; Elyamany et al., 2021), thereby inducing structural and functional changes in targeted regions (Madadi Asl et al., 2023; Herrmann et al., 2016; Hutt et al., 2018) potentially through diverse plasticity mechanisms (Shen et al., 2003; Pfister and Gerstner, 2006). This stimulation paradigm can entrain oscillations and elicit persistent after-effects lasting beyond the stimulation duration (Alagapan et al., 2016; Reato et al., 2010; Krause et al., 2022). Additionally, the efficacy of the entrainment and subsequent post-stimulation effects are state-dependent (Alagapan et al., 2016; Lefebvre et al., 2017), notably because of the competing influences of endogenous oscillations and tACS-induced forcing (Krause et al., 2022; Lefebvre et al., 2017). Multiple hypotheses for such persistent effects have been proposed, ranging from feedback reverberation (Alagapan et al., 2016; Park et al., 2018) to synaptic plasticity (Madadi Asl et al., 2023; Vogeti et al., 2022; Kromer and Tass, 2022; Schwab et al., 2021; Pfister and Tass, 2010). Yet, mechanisms remain poorly understood and outcomes are highly variable (Huang et al., 2017; Goldsworthy et al., 2016; Ridding and Ziemann, 2010).

Understanding the mechanisms underlying post-stimulation effects–critical for the clinical efficacy of tACS–remains challenging due to cellular heterogeneity. Numerous seminal studies show that neural responses to tACS, are influenced by biophysical properties like the membrane time constant (MTC) (Pariz et al., 2023), which shapes neuronal frequency selectivity and varies across cortical regions (Cheng and Lu, 2021; Moradi Chameh et al., 2021; Institute A. Dataset: Allen Institute for Brain Science, 2015). The MTC is a quantity that reflects the agility of neurons in response to time-varying stimuli (Cheng and Lu, 2021), and dictates their varied frequency selectivity (Pariz et al., 2023). The MTC varies significantly across cortical layers, and brain areas, ranging from a few to tens of milliseconds (Moradi Chameh et al., 2021; Institute A. Dataset: Allen Institute for Brain Science, 2015). Such variability has been shown to mediate selective, direction-specific synaptic plasticity under tACS (Pariz et al., 2023) and hence represents a promising candidate in supporting persistent post-stimulation effects. Indeed, stimulation-induced and MTC-dependent changes in neuronal spike timing, further modulated by endogenous oscillations, may solicit Hebbian spike timing dependent plasticity (STDP) to support changes in synaptic weights (Zaehle et al., 2010; Pariz et al., 2023). In this study, we investigated how low-amplitude sinusoidal stimulation (tACS) affects synaptic plasticity across neurons with heterogeneous MTCs and induce transient post-stimulation aftereffects. We explored two network states: a weak-coupling regime dominated by stimulation and a strong-coupling regime dominated by recurrent activity. Please note that in the Results section, we mainly focused on the weak coupling regime, and the strong coupling regime is presented and discussed in the Supplementary Material in details. We found that plasticity outcomes–and resulting changes in oscillatory power–were specific to stimulation amplitude and frequency, with excitatory–excitatory and inhibitory–excitatory connections playing key roles in generating persistent effects (Zaehle et al., 2010; Pariz et al., 2023; Kasten et al., 2016; Vossen et al., 2015). These findings emphasize the importance of accounting for biophysical diversity when designing stimulation protocols (Madadi Asl et al., 2023; Kromer and Tass, 2022; Kromer and Tass, 2024; Pfister and Tass, 2010).

## Results

Besides entrainment, which naturally occurs through the oscillatory modulation of targeted regions (Herrmann et al., 2016), one purpose of tACS is to yield persistent effects that outlast stimulation duration. Intuitively, this objective can not be fulfilled unless tACS changes some physiological characteristics of the area under intervention. While sufficiently large amplitude stimulation is capable of altering neuronal spiking activity (Pariz et al., 2023), the nature of the responses will also depend on the neurons’ heterogeneous biophysical attributes. Such a key attribute is the membrane time constant (MTC). The membrane time constant is a key parameter representing the agility of neurons in response to time-varying stimuli (Pariz et al., 2023; Cheng and Lu, 2021; Brette, 2015). Such wide heterogeneity in time scales translates into significant variability in neurons’ response to periodic stimulation: neuron spiking phase (in respect to the stimulation phase in which the neuron spikes) depends on the interplay between stimulation frequency and the neurons’ MTCs (Pariz et al., 2023). For instance, in the Leaky-Integrate and Fire (LIF) neuron model used in this study (see *Materials and methods*), differences in the spiking phase (i.e, $\Delta \phi \left(\tau_{m}\right)$ , where $\tau_{m}$ is the neuron MTC) resulting from a stimulation frequency $\omega_{s}$ between neurons with distinct MTCs can be translated into a difference in spike timing i.e., $\Delta T=\Delta \phi \left(\tau_{m}\right)/\omega_{s}$ . Such a difference in spike timing (Bi and Mm, 2001) has important implications for synaptic plasticity, stimulation-induced changes in synaptic weights, and their joint influence on endogenous oscillatory activity. Here we will explore the results of this interplay on neuronal population dynamics.

### Network properties and dynamic influenced by tACS

We built a network of 10,000 leaky integrate-and-fire (LIF) neurons, consisting of 8,000 excitatory (E) and 2,000 inhibitory (I) units, with a 10% connection probability and plastic synapses, to represent a cortical network (see *Materials and methods* and Table 1). Under these parameters, the network exhibits a Synchronous-Irregular (SI) balanced state (Brunel, 2000), characterized by a power spectrum peaked in the upper β band, with an endogenous frequency f∼30 Hz . We use f to denote the endogenous frequency, i.e., the frequency observed in the network in the absence of stimulation, and $\omega_{s}$ to refer to the exogenous frequency, i.e., the stimulation frequency.

**TABLE 1:** Parameters of the neuronal populations.

Parameters	Values	Description
$N_{E}$	8,000	Number of excitatory (E) neurons
$N_{I}$	2,000	Number of inhibitory (I) neurons
$P_{xy}$	10%, x,y∈[E,I]	Connectivity probability amongst neurons
$\tau_{m}$	$\mu_{\tau_{m}}=10$ , $\sigma_{\tau_{m}}=3\;ms$	Neuron membrane time constant (MTC)
$V_{\mathrm{rest}}$	−60 ±0.2 (mV)	Resting membrane potential
$g_{0}$	$1\times 10^{-3}$ (a.u.)	Initial Synaptic weight
$g_{0}^{E\to E}$	$g_{0}$ , $\sigma_{g}=0.1g_{0}$	Initial Synaptic weight amongst E to E neurons
$g_{0}^{E\to I}$	$g_{0}$ , $\sigma_{g}=0.1g_{0}$	Initial Synaptic weight amongst E to I neurons
$g_{0}^{I\to E}$	5 $g_{0}$ , $\sigma_{g}=0.1g_{0}$	Initial Synaptic weight amongst I to E neurons
$g_{0}^{I\to I}$	4 $g_{0}$ , $\sigma_{g}=0.1g_{0}$	Initial Synaptic weight amongst I to I neurons
$g_{\max}$	$2\times g_{0}$	Maximum value of synaptic weight
$E_{\mathrm{syn}}$	E=0 (mV) , I=−85 (mV)	Reversal potential
$t_{d}$	0.5–1 ms	Axonal delay
$\tau_{r}$	0.5 ms (AMPA), 0.5 ms ( ${\text{GABA}}_{a}$ )	Synaptic rise time constant
$\tau_{d}$	3 ms (AMPA), 5 ms ( ${\text{GABA}}_{a}$ )	Synaptic decay time constant
$v_{\mathrm{thr}}$	−54 (mV)	Threshold value
$\tau_{\mathrm{ref}}$	2 ms	Refractory time
$I_{\zeta }$	μ=5.5 (mV) and σ=1 (mV)	Mean input current and noise SD.
$A_{s}$	1 (mV)	Stimulation amplitude

To promote entrainment and improve the signal-to-noise ratio (i.e., contrast between endogenous oscillations and tACS), we set the system in a weak-coupling regime. In this configuration, the ratio of synaptic input to stimulation amplitude remains comparable, especially during the early stages of the simulation, before plasticity significantly alters connectivity. Although individual synaptic weights are small in this regime (see Table 1), the net synaptic current amplitude is comparable to stimulation-induced fluctuations: the average maximum synaptic current during population synchronous spiking is approximately 0.5 mV , with a standard deviation of ∼0.1 mV (see Supplementary Material for more details). In contrast, the strong-coupling regime emerges after ∼600 s of spontaneous network activity in the absence of stimulation. During this period, synaptic plasticity modifies the connectivity such that synaptic input dominates over stimulation amplitude. This regime avoids competition between recurrent synaptic inputs and stimulation-induced fluctuations (Krause et al., 2022; Lefebvre et al., 2017). We explored this condition in the Supplementary Material and found qualitatively similar results. In the rest of this study, we focus on results obtained under the weak-coupling regime.

**FIGURE 1:**
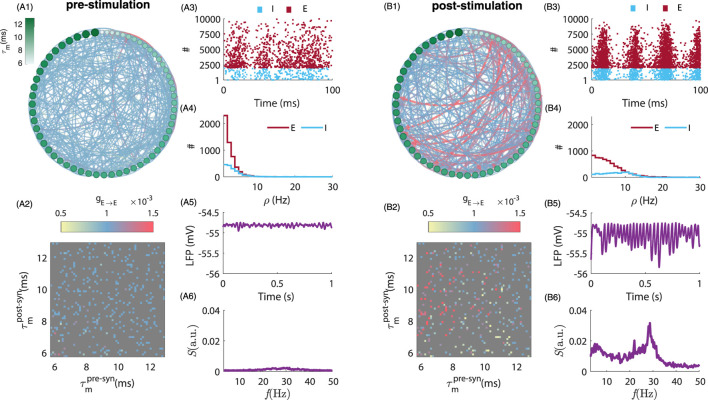
Comparison of neuronal network connectivity and dynamics before and after stimulation. **(A1,B1)** depict the pre- and post-stimulation population connectivity diagram, highlighting the changes in synaptic weights resulting from tACS. Here we plotted the connectivity amongst 60 randomly selected excitatory neurons during pre- (t<5 s) and post-stimulation (t>20 s) epochs, respectively. The neurons are sorted based on their MTC in a clockwise manner. The radius and colour of nodes indicated the change in the neuron’s MTC as the colorbar in **(A1)** The arrows indicate the connection from pre-to postsynaptic neurons. Synaptic weights are subjected to a Hebbian pair-based STDP (see 4). The arrows’ thickness and colour indicate the connection’s strength as colour-coded in **(A2,B2)** the corresponding synaptic weight matrices which are another representation of connectivity changes. The colorbar shows the strength of synaptic weights amongst pre- and postsynaptic neurons. **(A3,B3)** show the spiking activity of excitatory (E) and inhibitory (I) neurons in pre- and post-stimulation epochs, respectively. Note that the neurons’ spikes are plotted based on their MTC for each E (red dots) and I (blue dots) neuron, i.e., neurons with smaller MTCs have higher firing rates. **(A4,B4)** indicate neurons’ firing rates ρ in the pre- and post-stimulation epoch, respectively. The population shows synchronous irregular (SI) activity. Note that individual neuronal firing rates are smaller than the network’s endogenous oscillatory frequency. **(A5,B5)** show the LFP (see Equation 5) for pre- and post-stimulation epochs, respectively. **(A6,B6)** show the resultant power spectrum of population activity in pre- and post-stimulation epochs, respectively. Here, $\omega_{s}=25\;Hz$ , and $A_{s}=1\;\left(mV\right)$ . To plot the connectivity diagram **(A1,B1)** we used freely available software *Gephi* (Bastian et al., 2009).

We subjected this network to periodic stimulation of various amplitudes $\left(A_{s}\right)$ (Schwab et al., 2019), and frequencies $\left(\omega_{s}\right)$ , for a period of 15 seconds (simulation time, from t=5s to t=20s ). We then compared changes between the dynamics observed before stimulation (i.e., pre-stimulation) and after stimulation (i.e., post-stimulation) over epochs of 4 seconds. Specifically, we calculated the power spectrum over the pre-stimulation epoch (i.e., t=[1 5]s ), the stimulation epoch (i.e., t=[10 14]s ), as well as the post-stimulation epoch (i.e., t=[20.5 24.5]s ). The time intervals corresponding to each of those epochs have been selected to avoid any transient effects. To investigate the relationship between MTC heterogeneity and the persistence of stimulation-induced aftereffects, we plotted representative dynamics of the network in pre- and post-stimulation epochs in Figure 1 (See Supplementary Material for strong-coupling regime). We randomly selected 60 excitatory neurons and compared both network connectivity and the relative magnitude of synaptic weights between pre- and post-stimulation epochs in Figures 1A1,B1, respectively. Comparing these panels, one can readily notice stimulation-induced changes in synaptic weights and/or connectivity persisting well after stimulation offset. This effect was found to be mediated by variability in MTC. Corresponding synaptic weight matrices are plotted in Figures 1A2,B2, respectively. As shown in Figures 1A3,B3, the endogenous synchronous irregular activity present in the pre-stimulation period has been amplified in the post-stimulation epoch, accompanying a persistent increase in neuronal firing rates (Note that firing rates are lower than the network endogenous frequency as expected from irregular synchronous dynamics (Vogels and Abbott, 2005), i.e., the median of excitatory neurons firing rate is ∼0.5 Hz for pre-stimulation and ∼1.5 Hz for post-stimulation. See Figures 1A4,B4). The underlying population’s local field potential (LFP) (see Equation 5 in *Materials and methods*) also exhibits a significant increase in spectral power, especially salient at the endogenous (i.e., resonant) oscillation frequency and outlasting stimulation duration (see Figures 1A5,B5, also Figures 1A6,B6). We generalized these results in Supplementary Figures S2, S3, by choosing different distances to the threshold (by increasing the distance between resting and threshold potential) for each cell in the network, and by introducing heterogeneity in the threshold values. Note that this parameter change, even though it increased the amplitude of the oscillation, did not qualitatively change these results. The underlying mechanism behind this phenomenon may involve neurons being activated at different phases of stimulation, which induces selective synaptic weight modification and leads to amplified oscillatory activity. Further research is needed to explore the reasons behind this response, which are beyond the scope of this study.

### Post-stimulation aftereffects depend on stimulation parameters

**FIGURE 2:**
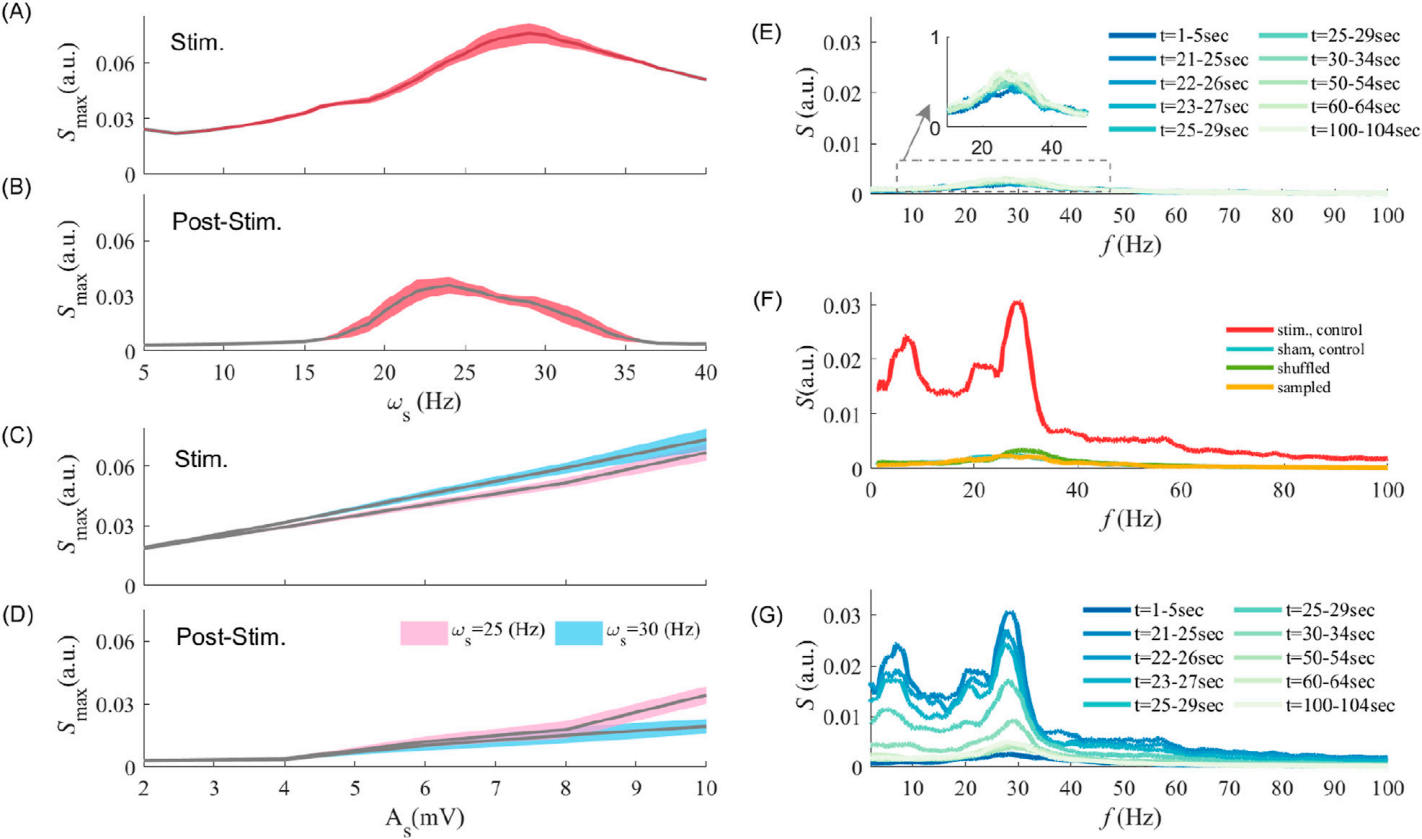
Interaction between stimulation frequency and amplitude in driving synaptic plasticity and post-stimulation aftereffects **(A,B)** show the maximum value of the LFP power spectrum at different stimulation frequencies during entrainment and post-stimulation epochs, respectively. Note that the maximum peak power may occur at different frequency other than the endogenous frequency, but fluctuates around the endogenous frequency f∼ 28 Hz . **(C,D)** show the maximum value of the LFP power spectrum while the amplitude of stimulation changes as the x-axis for entrainment and post-stimulation epochs, respectively, for $\omega_{s}=25$ , and  30 Hz . **(E–G)** display the power spectrum of LFP at different time points and situations. In **(E)** the stimulation is OFF, $\omega_{s}=0\;Hz,\;A_{s}=0\;\left(mV\right)$ , and the figure shows the power spectrum of population oscillation within 2 min (simulation time) of free evolution. **(F)** Power spectra obtained after shuffling synaptic weights (within each cell-type) and re-sampling synaptic weights from the same distribution, (within each cell-type). The synaptic weight matrix after turning off periodic stimulation suppresses spectral amplitude. Sham control condition refers to the case where there is no stimulation. The term sampled refers to the case where the neuronal population is built by randomly sampling synaptic weights from the same distribution. The power spectrum was computed at the end of the stimulation epoch (see *Materials and Methods*). **(G)** Illustrates the post-stimulation power changes observed at different time points. The colours, as the legend in **(E)** indicate the time intervals used to calculate the LFP power spectrum. In **(E–G)** the stimulation was ON over t∈[5 20)s with $A_{s}=1\;\left(mV\right)$ and $\omega_{s}=25\;Hz$ . The error bar, represented by the shaded area **(A–D)** denotes the standard deviation (SD) range around the trial-averaged values.

Having identified post-stimulation amplification in endogenous oscillations, we next evaluated how this phenomenon depends on stimulation parameters. In Figures 2A,B, we plot the peak LFP power spectrum for various stimulation frequencies, both during and after stimulation offset. Stimulating at frequencies ranging from $\omega_{s}=1\;Hz$ to $\omega_{s}=40\;Hz$ $\left(A_{s}=1\;\left(mV\right)\right)$ invariably increases LFP power during entrainment, especially for stimulation frequencies near the resonant endogenous frequency. The effect carried over to the post-stimulation epoch: as can be seen in Figure 2B, peak power remained high around the population endogenous frequency despite no stimulation being present, indicative of stimulation-induced engagement of synaptic plasticity. Optimal post-stimulation peak power was observed at a stimulation frequency of $\omega_{s}∼23\;Hz$ , which we note is different from the network endogenous oscillation observed before stimulation onset (f∼28 Hz) . This indicates that stimulation-induced changes in synaptic coupling might be higher at non-resonant frequencies, which possibly reflects the interaction of the neurons’ MTCs with the stimulation frequency.

Stimulation amplitude is also crucial to elicit - and possibly maintain - persistent entrainment and associated changes in synaptic coupling. We plotted in Figures 2C,D the peak LFP power as a function of stimulation amplitude (i.e., $A_{s}$ ) both during and after stimulation offset. Two stimulation frequencies (i.e., $\omega_{s}=25$ , and 30 Hz ) were considered as they both reside within the range of frequencies for which the effect of post-stimulation LFP power is significant (see Figure 2B). While peak LFP power increases linearly with stimulation amplitude during stimulation epochs (see Figure 2C), a thresholding effect can be observed in the post-stimulation period. Indeed, a minimum stimulation amplitude appears to be required to cause post-stimulation LFP power amplification (Figure 2D). These results indicate that a high stimulation amplitude is required to modulate the neurons’ membrane potential and spiking response, to cause changes in connectivity significant enough to yield observed post-stimulation effects. The difference in LFP spectral power between the two selected stimulation frequencies (i.e., $\omega_{s}=25$ and 30 Hz ) indicates that, despite expected stimulation-induced resonance (here at $\omega_{s}=\text{f}=30\;Hz$ , see Figure 2D), amplification may occur at different, non-resonant stimulation frequencies. We however, emphasize that stimulation-induced change in synaptic coupling may trigger shifts in endogenous oscillatory activity, causing the peak power to fluctuate around a frequency of f∼28 Hz ( ±1 Hz std.).

We further investigated whether and how MTC heterogeneity is involved in generating those results. Is the LFP power amplification observed post-stimulation due to a global, non-specific increase in synaptic coupling, or is it instead due to selective, MTC-mediated synaptic plasticity? To answer this question, we first explored the effects of STDP on post-stimulation power amplification. As shown in Figure 2E, in the absence of stimulation (i.e., sham; $A_{s}=0$ ) while the network remains exposed to STDP due to its own endogenous activity, no significant shift in LFP power can be observed.

Stimulation-induced amplification in post-stimulation power was found to rely heavily on selective synaptic modifications, i.e., synapse-specific directional changes resulting from periodic entrainment of neurons possessing distinct MTCs (Pariz et al., 2023). To expose the role of such selectivity, we randomly shuffled synaptic weights amongst neurons of the same cell-type while preserving their overall statistics (see *Materials and methods*). Figure 2F compares the spectral power obtained without stimulation (sham control; $A_{s}=0\;\left(mV\right)$ ) and post-stimulation (stim. Control; $\omega_{s}=25\;Hz$ , $A_{s}=1\;\left(mV\right)$ ) conditions with those obtained by shuffling and/or sampling synaptic weights randomly while preserving their respective distributions, within and between cell types. To do this, we first calculated the synaptic weight distribution amongst all synaptic types (i.e., E→E , E→I , and I→E ; Note that I→I remained unchanged). We next randomly *shuffled* synaptic weights in the network and examined whether post-stimulation oscillatory amplification could be observed over epochs of 4 s (no stimulation was applied during that period). As shown in Figure 2F, no post-stimulation increase in power could be observed, indicating that while displaying the same overall statistics (i.e., being shuffled, there are no changes in synaptic weights value and the distribution remains unchanged within each cell-type; See *Materials and methods*), selective plasticity between neurons with distinct MTCs is essential in generating amplified oscillation. We pushed the analysis further and *sampled* synaptic weights independently, only using the cell-type specific distributions calculated above (i.e., agnostic of the actual values of those weights). With this, the same result could be observed: in the absence of selectivity, post-stimulation oscillatory amplification vanishes.

Our results indicate that despite the significance of oscillatory amplification and its manifest reliance on MTC heterogeneity, all reported post-stimulation after-effects were found to be transient, as reported in several studies (Zaehle et al., 2010; Kasten et al., 2016; Vossen et al., 2015) and dissipate over time after stimulation is turned off. Upon stimulation offset, prevailing endogenous synchronous irregular activity engages STDP to bring synaptic connectivity back to baseline (see Figure 2G).

### Synaptic weights evolution depends on stimulation parameters and neurons’ properties

**FIGURE 3:**
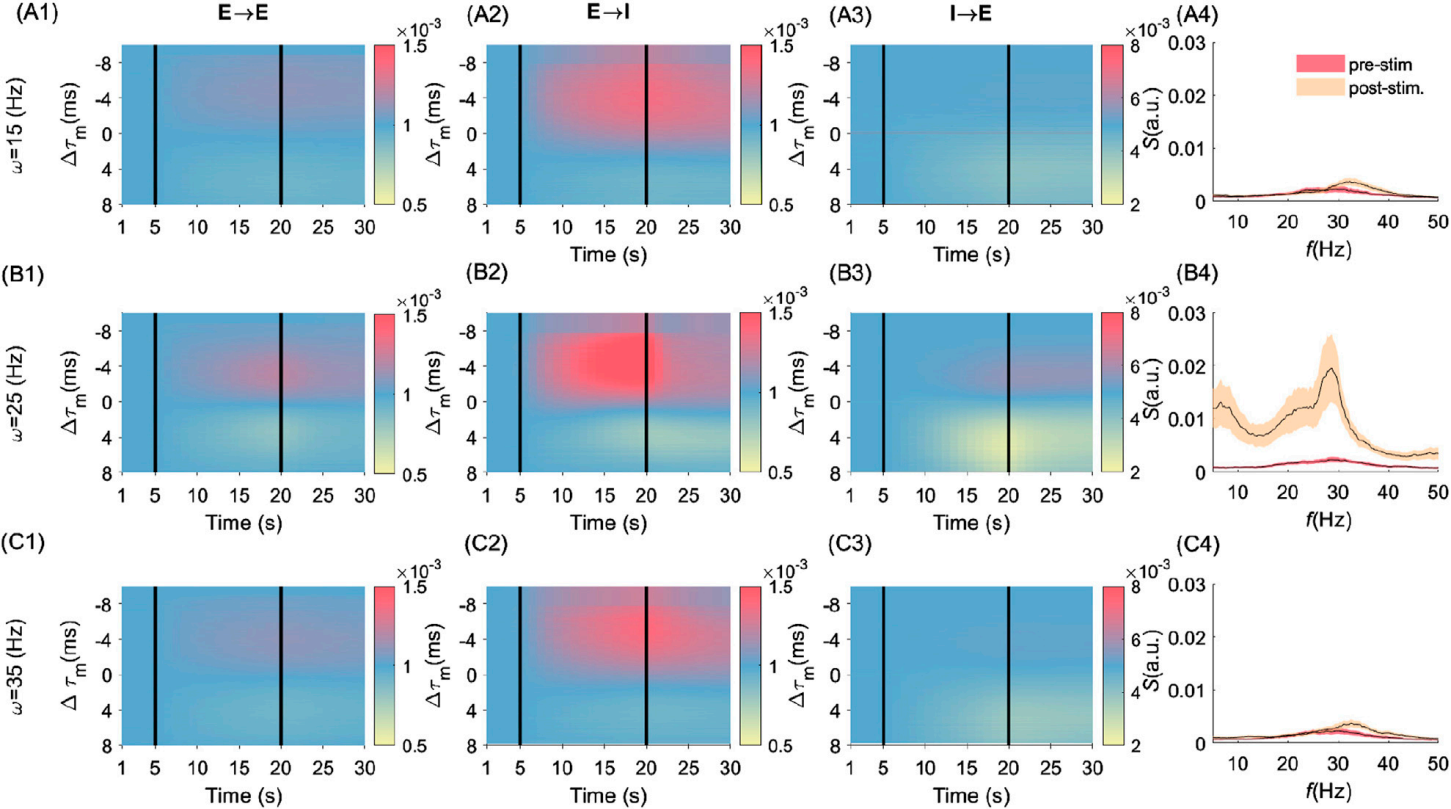
Frequency- and cell-type–specific effects of MTC heterogeneity on synaptic and spectral dynamics. Figure groups A (i.e., **A1-A4**), B (i.e., **B1-B4**), and C (i.e, **C1-C4**) are related to the stimulation frequencies $\omega_{s}=15,\;25$ , and 35 Hz , respectively. The heat-map plots show the dynamics of synaptic weights over time (x-axis) between synapses which we sorted according to their MTC difference (y-axis), $\Delta \tau_{m}=\tau_{m}^{\mathrm{pre}}-\tau_{m}^{\mathrm{post}}$ . Figures in each of the columns (first, second, and third column), from left to right, depict the evolution of the synaptic weights between E→E , E→I , and I→E , respectively, for the 30s (simulation time). Vertical lines in each panel divided the simulation into three epochs: the pre-stimulation (t=[1 5)s) , stimulation (t=[5 20)s) , and post-stimulation (t=[20 30)s) epochs. In the most right column, **(A4,B4,C4)** the power spectrum of neuronal population rhythm for pre- and post-stimulation epochs are plotted. For better comparison, we preserved the same y-axis range for all panels. The error bar, represented by the shaded area, denotes the standard deviation (SD) range around the trial-averaged values.

We examined the evolution of synaptic weights between all types of synapses in Figure 3 with respect to differences in MTCs, i.e., $\Delta \tau_{m}=\tau_{m}^{\mathrm{pre}}-\tau_{m}^{\mathrm{post}}$ . In Figure 3, we plot synaptic weights evolution for different stimulation frequencies i.e., $\omega_{s}=15\;Hz$ (Figures 3A1–A4), 25 Hz (Figures 3B1–B4), and 35 Hz (Figures 3C1–C4). These frequencies were selected to help the comparison between the dynamics and resulting plasticity at stimulation frequencies that either amplify the post-stimulation power (i.e., $\omega_{s}=25\;Hz$ ) and frequencies that do not ( $\omega_{s}=\;15,\;35\;Hz$ ; see Figure 2B). Although synaptic changes are noticeable in all of these cases, their relative magnitude was found to be highly frequency-specific. For instance, synaptic weights between excitatory and inhibitory neurons (i.e., E→I (Figure 3A2,B2,C2), display a broader range of synaptic modifications at $\omega_{s}=25\;Hz$ (Figure 3B2) compared to other frequencies (Figure 3A2,C2). This indicates that the stimulation frequency (∼25 Hz) , solicits MTC heterogeneity more strongly, leading to selective synaptic changes spanning a greater range of $\Delta \tau_{m}$ and stronger power amplification (see Figure 3A4,B4,C4). This is in contrast to Figure 3A2,C2 where synaptic weight changes were more selective for negative $\Delta \tau_{m}$ . The same effect could be observed for synapses between different cell types: selective modification observed amongst E→E and I→E synapses displayed a similar trend. Synaptic weight changes observed scaled with MTC mismatch as previously reported (Pariz et al., 2023), and further persisted over time after stimulation offset. In the last column, (A4), (B4), and (C4), for comparison purposes, we plotted the pre- and post-stimulation power resulting from each stimulation frequency used $\left(\omega_{s}=\;15,\;35\;Hz\right)$ .

### Influence of cell-type heterogeneity and synaptic plasticity on post-stimulation effects

**FIGURE 4:**
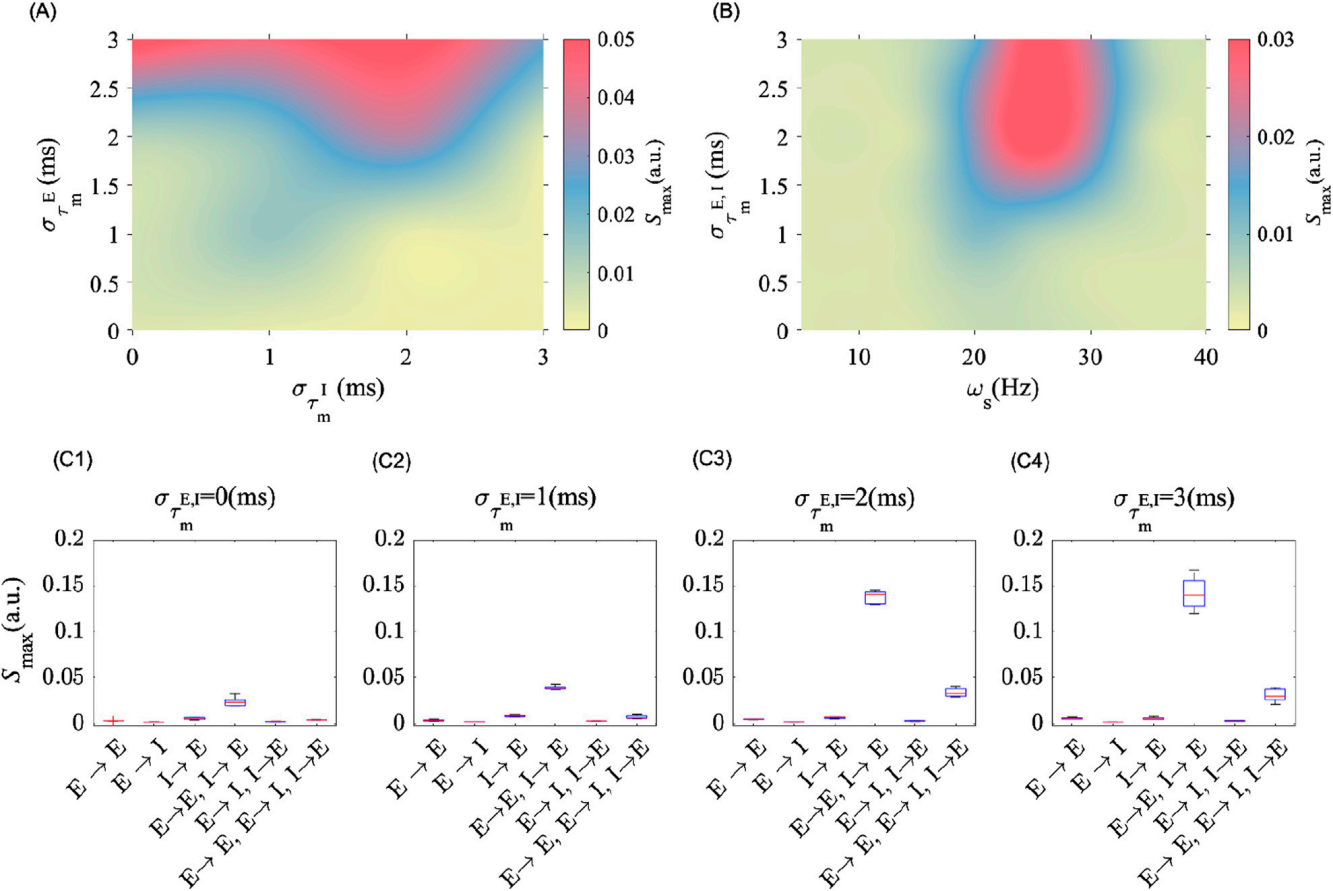
MTC heterogeneity amongst cell types modulates post-stimulation oscillation power. **(A)** Shows the peak spectral power in the post-stimulation epoch as the level of MTC heterogeneity of E (i.e., $\sigma_{\tau_{m}^{E}}$ ) and I (i.e., $\sigma_{\tau_{m}^{I}}$ ) cells is varied independently. The MTC distributions were drawn here from a Gaussian distribution, and $\sigma_{\tau_{m}^{E,I}}$ refers to the standard deviation. **(B)** Shows the peak spectral power in the post-stimulation epoch as a function of stimulation frequency $\left(\omega_{s}\right)$ and when the standard deviation $\left(\sigma_{\tau_{m}}\right)$ of MTC’s distribution of both E and I cells is varied. **(C1–C4)** show the changes in the peak spectral power in the post-stimulation epoch, while STDP is active only between the indicated groups of neurons along the horizontal axis, and for different values of $\sigma_{\tau_{m}^{E,\;I}}$ , respectively. In these plots $\omega_{s}=25\;Hz$ and $A_{s}=1\;\left(mV\right)$ .

Heterogeneity amongst and between different cell types, either excitatory or inhibitory, has different consequences on the post-stimulation power. To quantify this, we explored in Figure 4 the effects of cell-type MTC heterogeneity on post-stimulation LFP power. As shown in Figure 4A, MTC heterogeneity among excitatory neurons (i.e., $\sigma_{\tau_{m}^{E}}$ , along the horizontal axis) enhances post-stimulation power (i.e., ${\text{S}}_{\max}$ ), whereas increasing MTC heterogeneity among inhibitory neurons (i.e., $\sigma_{\tau_{m}^{I}}$ , along the vertical axis) abolishes the effects (See Figure 4A). The greater diversity observed among cortical inhibitory interneurons compared to excitatory neurons (Soltesz, 2006), may hinder stimulation effects and possibly prevent power amplification. However, it should be noted that the frequency of stimulation is another factor that determines the stimulation effects. We measured this in Figure 4B, where we varied the level of MTC heterogeneity of E and I neurons (i.e., both E and I neurons were assumed to express the same variation in MTC heterogeneity $\sigma_{\tau_{m}}$ ) and the frequency of stimulation. A similar increase in MTC variability of E and I neurons contributes to the induction of post-stimulation effects over a wider stimulation frequency range, i.e., [20 30] Hz . Having the same heterogeneity among inhibitory and excitatory neurons amplifies response power and therefore creates the necessary conditions for optimal synaptic weight changes, which ultimately leads to the amplification of oscillation power.

These results highlight the importance of considering plasticity among and between neuron subtypes. To investigate which synapses are more significantly involved in mediating the post-stimulation aftereffects, we applied periodic electrical stimulation on the same population at different degrees of MTC heterogeneity while selectively turning ON and OFF STDP amongst different cell types. This enabled the identification of synapses whose plasticity is more significantly solicited during stimulation. In Figures 4C1–C4, we show that plasticity between excitatory to excitatory neurons (that is, E→E ) and between inhibitory to excitatory neurons (that is, I→E ) is more involved in the amplification of the LFP power. The effect was also found to scale with the level of MTC heterogeneity across cell types (excitatory and inhibitory neurons) as of Figures 4C1–C4 where the post-stimulation power amplified as we increased the $\sigma_{\tau_{m}}^{E,\;I}$ . Introducing plasticity among inhibitory neurons, under the same conditions as previously considered, is found to suppress the amplitude of post-stimulation aftereffects (see and compare Supplementary Figures S1A, 2AB). These results suggest that blocking synaptic plasticity, whenever applicable, among synaptic subtypes may lead to a significant increase in post-stimulation power. Further investigations are required to determine the implications of MTC and cell-type specific synaptic blocking on tACS-induced aftereffects.

## Materials and methods

### Spiking neuron model

We modelled a population of excitatory and inhibitory Leaky-Integrate and Fire (LIF) neurons (Brette, 2015; Tuckwell, 2006). The differential equation for the evolution of the subthreshold membrane potential of each neuron is

$$\tau_{m}\frac{dv}{dt}=\left(V_{\mathrm{rest}}-v\right)+I_{\zeta }+I_{\mathrm{syn}}+I_{s},$$ (1)

where $\tau_{m}$ is the MTC, v is the membrane potential, $V_{\mathrm{rest}}$ is the resting membrane potential, and $I_{\zeta }$ represents an external current modelled here as white noise with a mean value of μ and a standard deviation σ . The term $I_{\mathrm{syn}}$ represents the synaptic current, while $I_{s}$ is the stimulation-induced current, which is here assumed to be a sinusoidal input (representing transcranial alternating current stimulation (tACS), (Krause et al., 2019; Krause et al., 2022; Schwab et al., 2021)), i.e., $I_{s}=A_{s}\sin\left(2\pi \omega_{s}t\right)$ , where $A_{s}$ and $\omega_{s}$ are the amplitude of the periodic signal, and the angular frequency respectively. When a neuron crosses the threshold value $v_{\mathrm{thr}}=-54\;\left(mV\right)$ , it spikes and its membrane potential resets to resting value $V_{\mathrm{rest}}=-60\;\left(mV\right)$ and remains there for $\tau_{\mathrm{ref}}=2\;ms$ representing the neuronal refractory period. Although having larger refractory periods alters the neurons’ firing, the results remain consistent (not shown). The parameters are in the physiological range (Gerstner et al., 2014) and summarized in Table 1. The total simulation time, unless otherwise stated, is 30 s, including pre-stimulation (sham epoch): t∈[0 5)s , stimulation epoch: t∈[5 20)s , and post-stimulation epoch: t∈[20 30]s (Extended stimulation periods did not show any significant difference. Data is not shown). The total synaptic current for neuron i is given by

$$I_{\mathrm{syn}}^{i}=\overset{N_{E}}{\underset{j=1}{\sum }}g_{ij}^{E}S_{ij}\left(t\right)\left(v_{i}-E_{\mathrm{syn}}^{j}\right)+\overset{N_{I}}{\underset{j=1}{\sum }}g_{ij}^{I}S_{ij}\left(t\right)\left(v_{i}-E_{\mathrm{syn}}^{j}\right)$$

where $g_{ij}^{E,I}$ are synaptic weights matrices associated with connections between either excitatory (E) and inhibitory (I) presynaptic neurons towards a postsynaptic neuron i . The sum is taken over $N_{E}$ excitatory and $N_{I}$ inhibitory presynaptic neurons over two nearest spike times. The reversal potential, $E_{\mathrm{syn}}$ , for E and I neurons are 0 (mV) and −80 (mV) , respectively. The synaptic response function $S_{ij}\left(t\right)$ for connections from neuron j to neuron i is modeled as

$$\begin{array}{ccc} S_{ij}\left(t\right) & = & \Lambda \;\exp\left(-\frac{t-t_{sp}^{j}-t_{d}^{ij}}{\tau_{r}}\right)-\exp\left(-\frac{t-t_{sp}^{j}-t_{d}^{ij}}{\tau_{d}}\right)\\ \Lambda & = & 1/\left({\left(\frac{\tau_{r}}{\tau_{d}}\right)}^{\frac{\tau_{r}}{\tau_{d}-\tau_{r}}}-{\left(\frac{\tau_{r}}{\tau_{d}}\right)}^{\frac{\tau_{d}}{\tau_{d}-\tau_{r}}}\right) \end{array}$$

where $t_{sp}^{j}$ is the spiking time of $j^{th}$ neuron, and $t_{d}^{ij}$ is the axonal delay between presynaptic neuron, j , and postsynaptic neuron, i . The $\tau_{r}$ and $\tau_{d}$ , are rise and decay synaptic time constants, respectively, associated with ${\text{GABA}}_{a}$ and AMPA receptors (see Table 1); (Gerstner et al., 2014).

Individual synaptic weights are randomly chosen from a normal distribution with mean and standard deviation as given in Table 1 (sham control). In *shuffled* (see Figure 2F), first we let the simulation run for 20s , and instantaneously shuffled the synaptic weights at the beginning of the post-stimulation epoch. We shuffled synaptic weights within each synapse category (i.e., the synaptic weights among E and I neurons). In the *sampled* case (see Figure 2F), we took the following procedure: We let the population in *stim. Control* evolve for 20s (5s pre-stimulation, and 15s stimulation epochs). We then calculated the distribution of the synaptic weights at the end of the stimulation epoch. Then we used these distributions to randomly sample synaptic weights within each synapse category ( E→E , E→I , and I→E ) using this fitted distribution. To fit the distribution, we used *cftool* package in MATLAB. We seek any function that fits the data with R−squre>0.95% . A representation of this distribution is being shown in S4. These tests demonstrate that while the overall distribution of synaptic weight may remain intact through shuffling or sampling, selective modification is essential for inducing post-stimulation aftereffects.

### Spike timing dependent plasticity (STDP)

Plasticity in our population amongst connected neurons is modelled using Hebbian pair-based spike-timing dependent plasticity (Choe et al., 2013; Gütig et al., 2003; Sjöström et al., 2010). To avoid biased synaptic changes (i.e., preferential LTP/LTD.) we chose a symmetric STDP Hebbian learning rule (Bi and Mm, 2001; Gütig et al., 2003; Sjöström et al., 2010). The synaptic weight dynamics in our model follows the below equations:

$$\begin{array}{ccc} \Delta g & = & \left\{\begin{array}{cc} A_{+}\left(1-g/g_{\text{max}}\right)\exp\left(-\Delta T/\gamma^{+}\right),\; & \text{if }\Delta T\geq 0.\\ -A_{-}\left(g/g_{0}\right)\exp\left(\Delta T/\gamma^{-}\right),\; & \text{if }\Delta T<0. \end{array}\right.\\ g & = & g+\Delta g \end{array}$$ (2)

The $\gamma^{+}$ and $\gamma^{-}$ are STDP decay time constants. $\Delta T=t_{sp}^{\mathrm{post}}-t_{sp}^{\mathrm{pre}}-t_{d}$ is the time difference between the spiking time of post- and presynaptic neurons, and $t_{d}$ is delay between presynaptic and postsynaptic neurons. Whenever ΔT is positive (negative), the synaptic weight between *presynaptic* to *postsynaptic* neurons gets potentiated (depressed). The constant $g_{\text{max}}$ denotes the maximum achievable synaptic weight, while $g_{0}$ denotes the initial synaptic weight, taken from a narrow Gaussian distribution across all synaptic connections before learning (see Table 1).

Baseline synaptic coupling and threshold were selected to set the network in a weak-coupling regime, sub-threshold regime, in which an isolated presynaptic spike does not guarantee postsynaptic firing. This regime achieved by choosing the synaptic weight from a narrow distribution (see Table. 1) and at the early stage of simulation. Despite weak synaptic coupling, the afferent synchronous synaptic input each neuron receives from the rest of the network is comparable to the stimulation amplitude, i.e., the average of maximum synaptic input (at the onset of every synchronous spiking activity) and its standard deviation is ∼0.5 (mV) and ∼0.1 (mV) , respectively. In the strong-coupling regime, which the network reaches after 600 s of simulation time (in the absence of stimulation), the average maximum synaptic input and its standard deviation reach approximately ∼1.5 (mV) and ∼0.45 (mV) , respectively. Throughout this report, we used Equation 2 for synaptic modification, and our choice of STDP parameters are $A_{+}=2A_{-}=4\times 10^{-4}$ , $g_{\max}=2g_{0}$ and $\gamma_{\pm }=10\;ms$ .

### Network model

We modelled a randomly connected sparse network of 10,000, LIF neurons (see Equation 1) with a 4:1 ratio of E (8000) and I (2000) neurons with a fixed connection probability of 0.1 (Vogels and Abbott, 2005; Bryson et al., 2021; Campagnola et al., 2022). To balance physiological relevance and computational tractability for the network sizes we used the LIF neurons model (Burkitt, 2006). The synaptic weights and other neurons’ parameters have been selected within the reported physiological range (Campagnola et al., 2022) to be in line with previous studies on LIF cortical network models (see (Burkitt, 2006; Kobay and ashi, 2009; Brunel and Wang, 2003) and references therein), and are further summarized in Table 1. To study the effect of MTC heterogeneity, we randomly sampled neuronal MTCs $\left(\tau_{m}\right)$ from Gaussian distribution with $\mu_{\tau_{m}}=10\;ms$ , and $\sigma_{\tau_{m}}=3\;ms$ unless otherwise specified. The resulting population exhibits a synchronous irregular activity (SI) (i.e., see Figure 1A3).

### Power spectral analysis

To perform spectral analysis of the network’s mean activity, we first calculated the local field potential (LFP), $\bar{V}$ as the weighted ensemble average of the membrane potential i.e., (Herrmann et al., 2016; Hutt et al., 2018; Lefebvre et al., 2017; Mazzoni et al., 2015; Bazhenov et al., 2001),

$$\bar{V}=\frac{0.8}{N_{E}}\overset{N_{E}}{\underset{i=1}{\sum }}V_{E}^{i}+\frac{0.2}{N_{I}}\overset{N_{I}}{\underset{i=1}{\sum }}V_{I}^{i},$$ (3)

where the relative proportion of excitatory (0.8) versus inhibitory interneurons (0.2) cells is taken into consideration. The power spectral density of $\bar{V}$ was averaged over 10 independent trials in which the same stimulation protocol is applied, but using different baseline connectivity, synaptic weights, and noise realizations. For the purpose of Figures 1, 2, 4, we further took the average of the power spectral density with a moving average window (with MATLAB *smooth* function) with σ=1.5 Hz , that provided us with a smoothed power-frequency curves (for instance, see Figure 2B6).

## Discussion

To better understand the mechanism underlying post-stimulation amplification in oscillatory activity observed in experiments (Alagapan et al., 2016; Clancy et al., 2022), we extended the framework of selective STDP (Pariz et al., 2023) in a synchronous, sparsely connected neuronal network of heterogeneous spiking neurons. We computationally showed that in the presence of endogenous synchronous activity, near-resonant periodic stimulation may amplify post-stimulation power through selective synaptic changes, whose magnitude and direction rely on intrinsic differences in MTC. Stimulation at the near-resonant frequency was found to engage STDP so that the population expresses higher endogenous oscillatory power (see Figure 2B), resulting in transient yet prolonged overlasting effects. We confirmed that selective, directional changes in synaptic coupling - both within and between cell types - are responsible for such amplification, while any shuffled, randomly assigned synaptic weights, or intrinsic synaptic weight changes in the absence of stimulation, are insufficient for generating aftereffects on their own (see Figures 2E–G). The level of heterogeneity in neuronal MTC was found to determine the efficacy of stimulation on post-stimulation power magnitude and duration (see Figure 4A). Indeed, in a homogeneous network (i.e., where the MTCs are identical), neurons respond similarly to a given stimulus. Because of the symmetric nature of our STDP rule, such homogeneity might prevent stimulation-induced synaptic plasticity, even in the presence of noise. This means that a minimum level of heterogeneity is essential for pushing STDP in one direction or another, especially while interacting with time-varying inputs. Taken together, these results echo previous studies (Madadi Asl et al., 2023; Kromer and Tass, 2022; Pfister and Tass, 2010) by revealing one potential mechanism behind the effectiveness of tACS for therapeutic purposes, specifically the stabilization of stimulation effects on neural dynamics and connectivity. We argue that heterogeneity in neuronal time scales represents a dominant contributor mediating tACS efficacy, affirming the neurophysiological bases of persistent entrainment towards the development and/or optimization of clinical interventions. The results were qualitatively similar in both early stage of simulation and in the late stage where the synaptic weights modification, in the absence of stimulation, reaches a steady state. Further results for the latter case can be found in the Supplementary Material section. In short, we showed that even in the steady state, where the synaptic input currents are larger with respect to stimulation amplitude (up to three times the stimulation amplitude; i.e., strong-coupling regime), the post-stimulation aftereffects still depend on stimulation frequency and amplitude, as well as the MTC heterogeneity level (See. S6 and S9).

It should be noted that the results we report here extend to a broad range of endogenous frequencies. For instance, networks expressing oscillations within the alpha range may need different stimulation frequencies to solicit selectivity in synaptic plasticity (Lefebvre et al., 2017). This has important implications given the broad variety of frequencies characterizing oscillopathies (Takeuchi and Berényi, 2020; Hammond et al., 2007; Uhlhaas and Singer, 2013), where tACS hold promise to perturb pathological rhythms to unveil the mechanisms and potentially treat neurological and/or neuropsychiatric disorders. Interestingly, while stimulating at resonant/endogenous frequency expectedly yields higher entrainment (Lefebvre et al., 2017) (see Figure 2A), this does not always accompany significant post-stimulation aftereffects (see Figure 2B). We point out that our simulations also support a state-dependent dependence on stimulation efficacy. Indeed, weak background synaptic activity resulted in a high signal-to-noise ratio i.e., stimulation-induced modulation in neuronal membrane potential was significant enough to trigger depolarization and hence recruit STDP. In the presence of strong synaptic activity, however, the effects may fade away (Krause et al., 2022; Lefebvre et al., 2017). We also emphasize that to engage populations expressing a wide range of MTC, stimulation amplitude must scale accordingly, potentially influencing neuronal firing rates (Pariz et al., 2023). The precise relationship between stimulation frequency, synaptic plasticity, and persistent entrainment remains to be fully explored.

Nonetheless, our model suffers from limitations. First, we considered a neuronal network with random local (i.e., close spatial proximity where axonal conduction delays are considered small) connectivity, among cell types ( E→E , E→I , I→E , and I→I ). The more realistic network as observed experimentally (Rubinov et al., 2011) has a different connectivity distribution which should be considered in later investigations. Note that changes in connectivity could lead to different axonal delay distributions among neurons which then may influence the synaptic plasticity dynamics (Madadi Asl et al., 2018). Second, the Hebbian pair-based STDP rule, and our assumption that all synapses obey the same rule, are limiting the generality of our results. Future investigations need to consider the large variety of synaptic plasticity mechanisms between cell types (Abbott and Nelson, 2000; Caporale and Dan, 2008) and the possible heterogeneity in STDP parameters. Note that introducing both Hebbian and anti-Hebbian plasticity for efferent inhibitory synapses (i.e., I→I and I→E ) (Abbott and Nelson, 2000; Caporale and Dan, 2008; Dan and Poo, 2004; D’amour and Froemke, 2015) yields qualitatively similar results (strong entrainment is observed around $\omega_{s}=30\;Hz$ and the peak power can be observed for frequencies between $\omega_{s}∼20-30\;Hz$ . See Figures 2A,B), yet the amplitude is changed (see Supplementary Figure S1A). These results showcase the importance of synaptic dynamics on the emergence of oscillatory activity in recurrent neural networks and warrant further investigation.

Synaptic plasticity selectivity is not limited to heterogeneity in MTC: other sources of heterogeneity, such as the resting membrane potential, rheobase, and/or spiking threshold, may promote cell-to-cell differences in spike timing. Lastly, we have mapped neurons’ MTC using a normal distribution, whose variance $\sigma_{\tau_{m}}$ (i.e., scaling with the degree of heterogeneity) alters the number of synapses that can be effectively modified by stimulation. However, similar to natural phenomena, the MTC distribution may be better fitted using a gamma or lognormal distribution (Pariz et al., 2023; Limpert et al., 2001; Buzsáki and Mizuseki, 2014).

Another limitation arises from our choice of using the same MTC distribution for both excitatory and inhibitory neurons. This choice was motivated by the need to balance physiological relevance and computational tractability - as well as limiting the dimensionality of the analysis. While the introduction of cell-type specific MTC distributions would certainly influence our results, we note that by construction, excitatory and inhibitory cells in our network already display differences in firing rates (e.g., see Figure 1). Further investigations are warranted to thoroughly examine such additional sources of heterogeneity. We however, hypothesize that as long as the overall activity of the neuronal population remains within an oscillatory synchronous irregular state, characterized by a low level of coherency, similar results would be observed.

## Conclusion

Brain stimulation techniques offer invasive and non-invasive treatments for brain-related disorders. The promising results in the application of these techniques attracted a wide range of interdisciplinary researchers to investigate the response of brain cells to these interventions and devise more effective and reliable methods. Towards this goal, our study expanded the knowledge of how periodic stimulation may enhance and stabilize post-stimulation effects. Our results emphasize the importance of neural timescale variability in the interaction between synaptic plasticity and tACS. Overall, our results elucidate one potential mechanism by which tACS affects neural population connectivity, and conditions under which such intervention can lead to amplified, overlasting effects.

## Data Availability

The datasets presented in this article are not readily available because our study is a modeling study in which we simulated neuronal activity and analyzed the results. The codes we used are publicly available on GitHub https://github.com/arefpz/neuronal_population. Requests to access the datasets should be directed to pariz.aref@gmail.com.
