# Supplementary material for: Amplifying post-stimulation oscillatory dynamics by engaging synaptic plasticity with transcranial alternating current stimulation
Source: Front Netw Physiol. 2025 Jul 18;5:1621283. doi: 10.3389/fnetp.2025.1621283 (PMC12314431; doi:10.3389/fnetp.2025.1621283)
Supplement: Supplementary file 1 [file Supplementaryfile1.pdf]

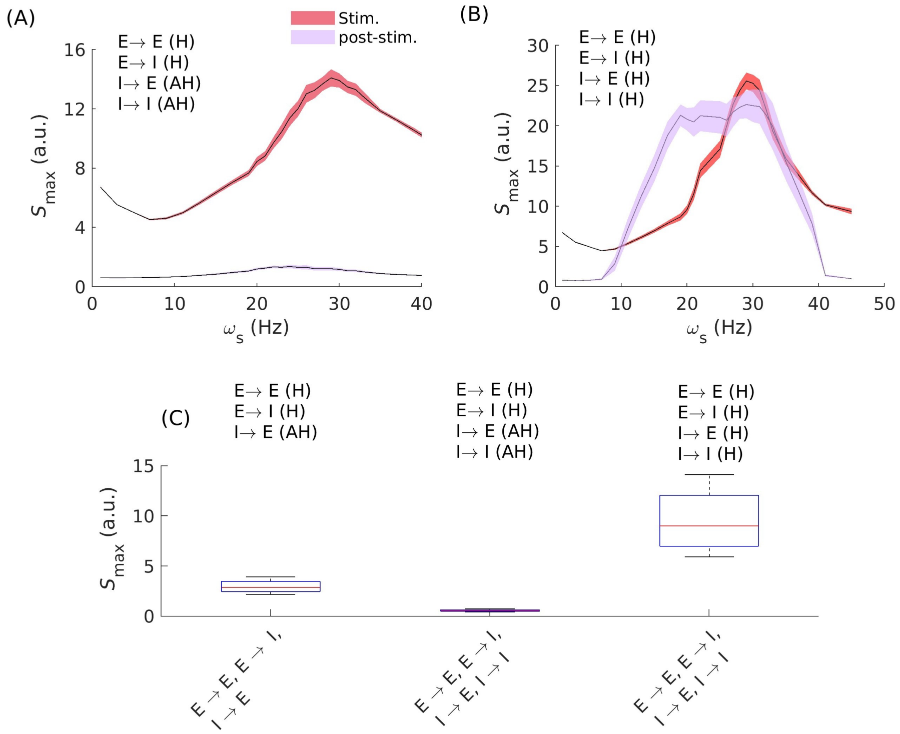

Fig S1. Anti-Hebbian and Hebbian STDP effects on post-stimulation power spectrum in weak-coupling regime. (A) shows the power spectrum of stimulation and post-stimulation epochs when the synaptic weights are modified with the Hebbian (H) STDP rule for efferent excitatory synapses and anti-Hebbian (AH) rule for efferent inhibitory synapses. Note that here we allowed the plasticity dynamics over $I \to I$ connections. (B) shows the power spectrum of stimulation and post-stimulation epochs, when the Hebbian STDP rule is active for all efferent synapses from excitatory and inhibitory neurons. (C) Comparison of the post-simulation power spectrum for three cases, as noted on the x-axis and text above each category. The results showcase the effects of synaptic plasticity on post-stimulation power modification. Introducing the plasticity among inhibitory neurons reduced the post-stimulation power amplitude in comparison to Fig. **??**, where the $I \to I$ plasticity was absent. The STDP type (Hebbian, anti-Hebbian) not only affects the post-stimulation epoch power amplitude but also affects the entrainment power amplitude (see A and B), which needs further investigation for more clarification.

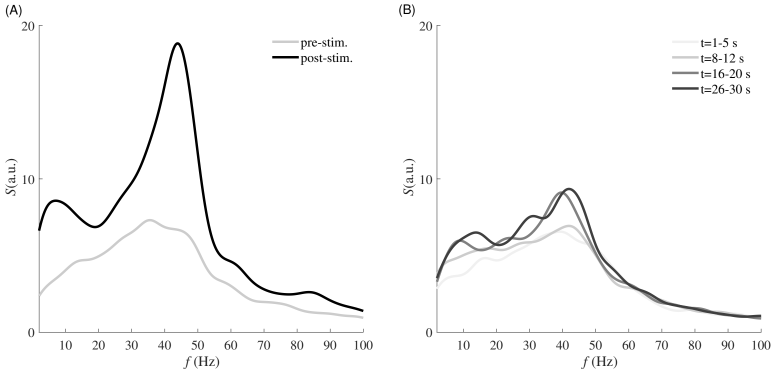

Fig S2. Effect of heterogeneity in neurons' spiking thresholds. By introducing spiking threshold heterogeneity in both excitatory and inhibitory cells, the population oscillates at a different endogenous frequency ($f = 38\ Hz$). Applying stimulation at the peak frequency, (i.e., $\omega_s \approx f = 35\ Hz$), and , $A_s = 1\ (mV)$ for 60 s enhanced the oscillation amplitude. For comparison purposes, in (A) and (B), we plotted the power spectrum with and without threshold distribution, respectively. The threshold values were sampled from a normal distribution with $\mu_{thr} = -54\ (mV),\ \sigma_{thr} = 2\ (mV)$.

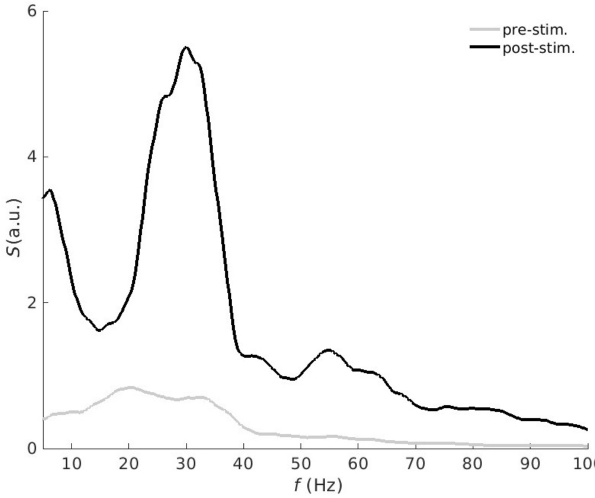

Fig S3. Larger distance to threshold values has no significant impact on the pre- and post-stimulation oscillation amplitude (weak-coupling regime). The population oscillation frequency in pre- and post-stimulation epochs is shown as grey and black lines, respectively, when the distance to the threshold has increased to 16 (mV). To push the neurons toward the spiking threshold region, we changed the input current in Eq. **??** to 15.5 (mV). In this case, the ratio of stimulation amplitude and distance to the threshold is reduced to ~ 0.06 (originally was ~ 0.17).

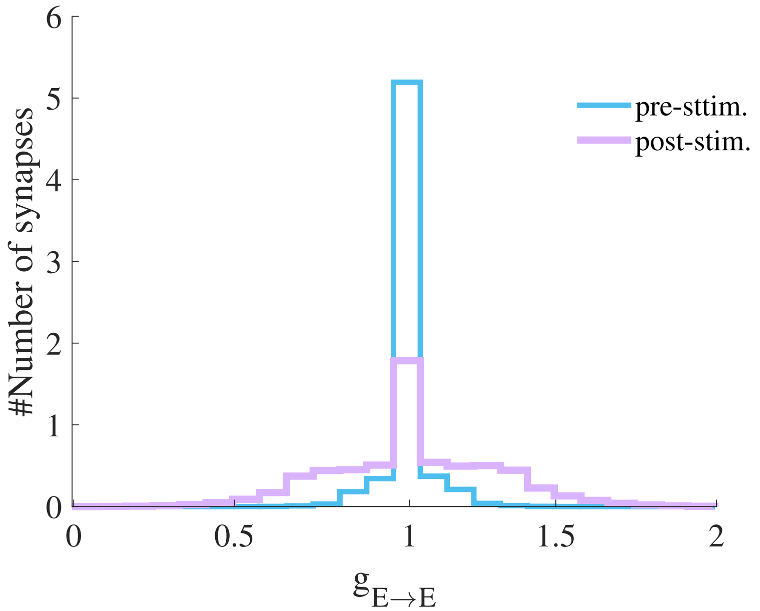

Fig S4. Synaptic weight distribution in pre- and post-stimulation epochs in weak-coupling regime. The distribution of synaptic weight just before the onset of stimulation ($t = 5s$) and just after stimulation ($t = 20s$) among excitatory neurons, is plotted. We used multiple functions (i.e. Double Gaussian) to fit the post-stimulation synaptic weights for each of synaptic weight categories ($E \to E$, $E \to I$, and $I \to E$) with $R - squre > 0.95$ and used these function to sample the synaptic weight randomly (see *Materials and methods* section).

Strong-Coupling regime

In this study, we investigated the effects of stimulation frequency and amplitude on the power spectrum of the post-stimulation LFP. In the main text, we show that in a weak-coupling regime, where the majority of synaptic weights are far from their maximum (i.e. g_{max}) and minimum values (i.e. $0.01g_0$), the stimulation can selectively change the synaptic weights, inducing amplified post-stimulation power. This supplementary section will explore the network's response in a strong-coupling regime to periodic stimulation.

By allowing the network to evolve for 600 seconds (simulation time), on average, the net synaptic input current each neuron receives from the rest of the network reaches up to about three times the input current it received at the early stages of the simulation. We refer to this state as a strong-coupling regime, where the neuron's input current exceeds the stimulation amplitude. In this state, the synaptic weights continue to change, but the overall dynamics of the network have reached a stationary state. We define the sum of synaptic weights for each synapse (i.e. $E \rightarrow E$) at each time point as our order parameter, illustrating the steady-state dynamics.

In Fig. S5, we plotted the network properties similar to Fig. **??**. There are qualitative differences between these two regimes, but overall, both are showing the effects of stimulation on the synaptic weights modifications, firing rate, and power spectrum just after stimulation turned OFF. By comparing Fig. S5 (A1) and (B1) with Fig. **??** (A1) and (B1), one can notice the difference in the selective changes over network connections. Although the changes in the strong-coupling regime are less than in the weak-coupling regime, one can still notice connections that have been weakened or strengthened. This reflects the fact that in this regime, the stimulation was not able to engage a majority of the neurons, due to a small ratio of stimulation amplitude to neurons' synaptic input. The network in steady state and pre-stimulation epoch shows higher activity compared to the weak-coupling regime (see Fig. S5(A3) and Fig. **??** (A3) for compar-

ison). However, the amplification of the LFP power is still noticeable in this state (see Fig. S5 B6 and A6).

Figures S6 (A) and (B) explore the effects of the stimulation on the network activity in the strong regime in pre- and post-stimulation epochs, respectively. Fig. S6 (C) and (D) show the power spectrum in the pre- and post-stimulation epochs, evaluated at different time points, respectively. Figure S6 (E) compares the power spectrum of pre-stimulation and post-stimulation epochs, and the effects of shuffling the synaptic weight in each synaptic subtype category (i.e. $E \rightarrow E$, see *Materials and Methods*) in the post-stimulation epochs that qualitatively are similar to Fig. **??**.

The time evolution of the synaptic weights is shown in Fig. S7 for three different synaptic subtypes ($E \rightarrow E$, $E \rightarrow I$, and $I \rightarrow E$) and stimulation frequencies, $\omega_s = 25,\ 35$, and $45\ Hz$, arranged in different rows. The resulting power spectrum for the pre-stimulation and post-stimulation epochs is displayed in the right column (A4, B4, and C4). In the strong-coupling regime, due to intense interference between synaptic input current and stimulation amplitude, the phase response specificity of neurons (based on their MTC) is not as sensitive as it was in the weak-coupling regime, leading to a qualitatively similar trend in synaptic modification across all $\Delta\tau_m$.

Similar to the weak-coupling regime, the post-stimulation aftereffects depend on the stimulation frequency. Just after the end of stimulation at $\omega_s = 35\ Hz$, the population activity shows a high power spectrum; however, a few seconds later, it diminishes and drops to a power level even lower than that of the pre-stimulation epoch (see the curve for $t \in [1350\ 1355]s$). In contrast to the weak-coupling regime, where the transient post-stimulation power spectrum returns to pre-stimulation levels, the stimulation suppresses the power spectrum even lower than the pre-stimulation level. This phenomenon raises questions regarding the application of brain stimulation in different brain states (i.e., resting), which requires further investigation.

At the beginning of the simulation, we selected the synaptic weights in

each subcategory (i.e. $E \to E$) from a narrow distribution (see Table **??**). Over time, the synaptic weights have been adjusted and reached a steady state. We plotted the distribution of synaptic weights in each subcategory in Fig. S8. The sum of the synaptic weights is represented as a black line. These plots demonstrate a rapid transient change in the distribution of the sum of the synaptic weights due to the application of stimulation over $t \in [600\ 660]s$. In the post-stimulation epoch, the sum of the synaptic weights exhibits fast and slow transitions.

Both weak and strong-coupling regimes exhibit MTC and frequency dependency in LFP power during the post-stimulation epoch. In Fig. S9, we reproduce the same results shown in Fig. **??**, illustrating that the distribution of MTC and the frequency of stimulation can affect the level of LFP power amplification. The impact of synaptic weight modification across different types of synapses is illustrated in Fig. S9 (C1) to (C4). In contrast to the weak-coupling regime, the strong-coupling regime displays efficient LFP power amplification due to plasticity among $E \to E$, $E \to I$, and $I \to E$ (see and compare Fig. S9 and Fig. **??**).

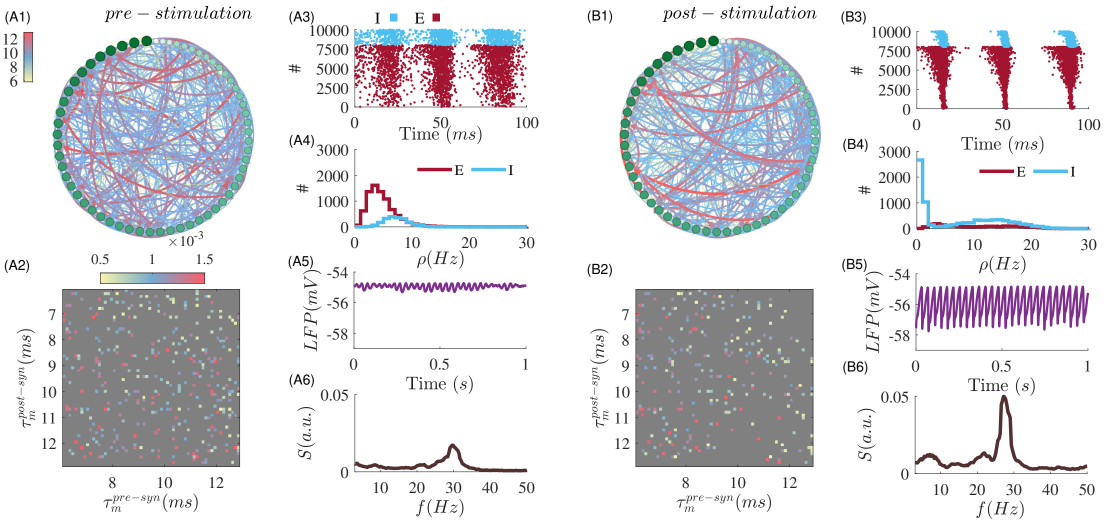

Fig S5. Amplified post-stimulation endogenous oscillations (strong-coupling regime). (A1) and (B1) depict the pre- and post-stimulation population connectivity diagram, highlighting the changes in synaptic weights resulting from tACS. Here, we plotted the connectivity amongst 60 randomly selected excitatory neurons during pre- ($t = 600$ s) and post-stimulation ($t = 665$ s) epochs, respectively. The neurons are sorted based on their MTC in a clockwise manner. The radius and colour of nodes indicated the change in the neuron's MTC as the colorbar in (A1). The arrows indicate the connection from pre- to postsynaptic neurons. Synaptic weights are subjected to a Hebbian pair-based STDP (see Fig. **??**). The arrows' thickness and colour indicate the connection's strength as colour-coded in (A2) and (B2), the corresponding synaptic weight matrices, which are another representation of connectivity changes. The colorbar shows the strength of synaptic weights amongst pre- and postsynaptic neurons. (A3) and (B3) show the spiking activity of excitatory (E) and inhibitory (I) neurons in pre- and post-stimulation epochs, respectively. Note that the neurons' spikes are plotted based on their MTC for each E (red dots) and I (blue dots) neuron, i.e., neurons with smaller MTCs have higher firing rates. (A4) and (B4) indicate neurons' firing rates ρ in the pre- and post-stimulation epochs, respectively. The population shows synchronous irregular (SI) activity. Note that individual neuronal firing rates are smaller than the network's endogenous oscillatory frequency. (A5) and (B5) show the LFP (see Eq. **??**) for pre- and post-stimulation epochs, respectively. (A6) and (B6) show the resultant power spectrum of population activity in pre- and post-stimulation epochs, respectively. Here, $\omega_s = 35$ Hz, and $A_s = 1$ (mV). To plot the connectivity diagram (A1 and B1), we used freely available software *Gephi* [**?**].

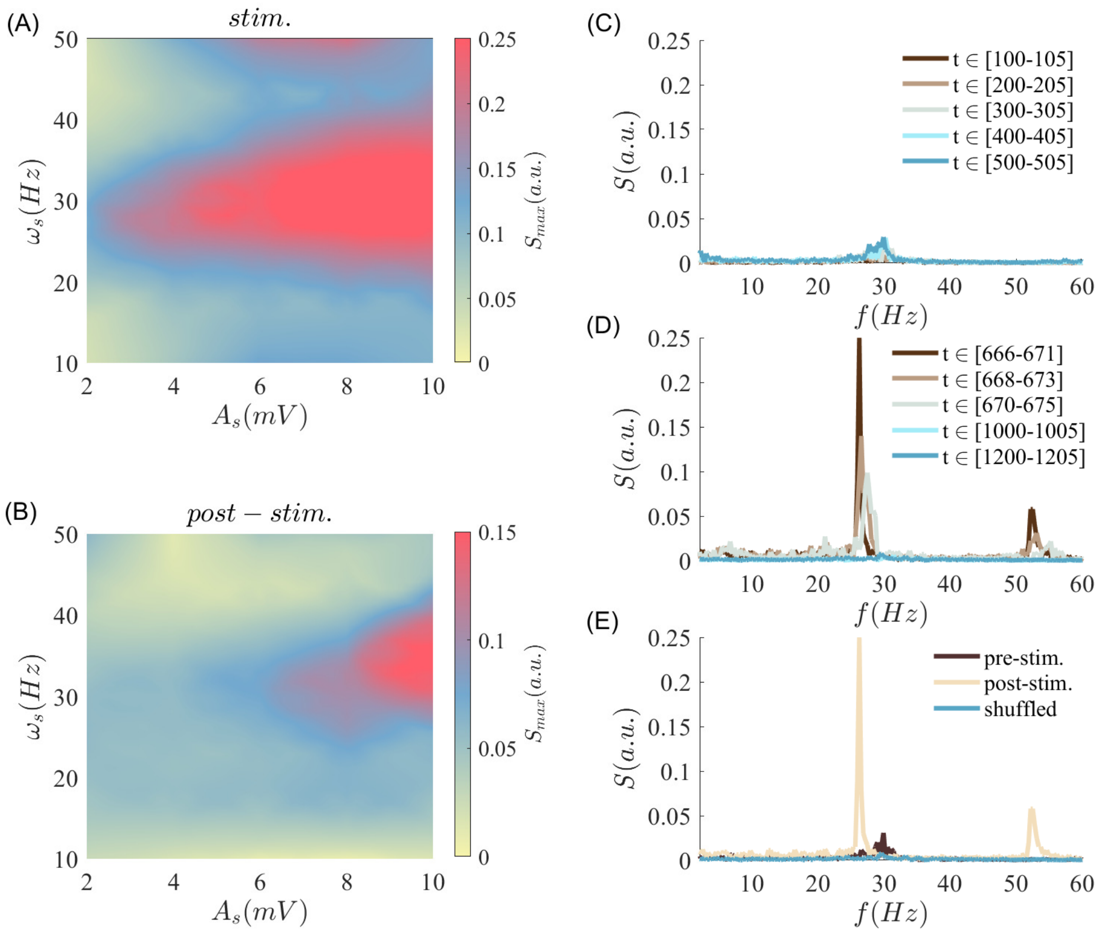

Fig S6. Effect of stimulation frequency and amplitude on post-stimulation activity in strong-coupling regime. (A) and (B) show the maximum value of the LFP power spectrum at different stimulation frequencies (as of the y-axis) and amplitudes (as of the x-axis) during entrainment and post-stimulation epochs, respectively. Note that the maximum peak power may occur at a frequency other than endogenous frequency, but fluctuates around endogenous frequency $f \sim 28Hz$. Here $A_s = 1$ (mV). (C) and (D) show the power spectrum of LFP at different time points in pre-stimulation and post-stimulation epochs, respectively. In (E), the power spectrum of population oscillation for pre- and post-stimulation epochs is depicted. In the case of a shuffled synaptic weights matrix in the post-stimulation epoch, the power spectrum does not show any amplified power.

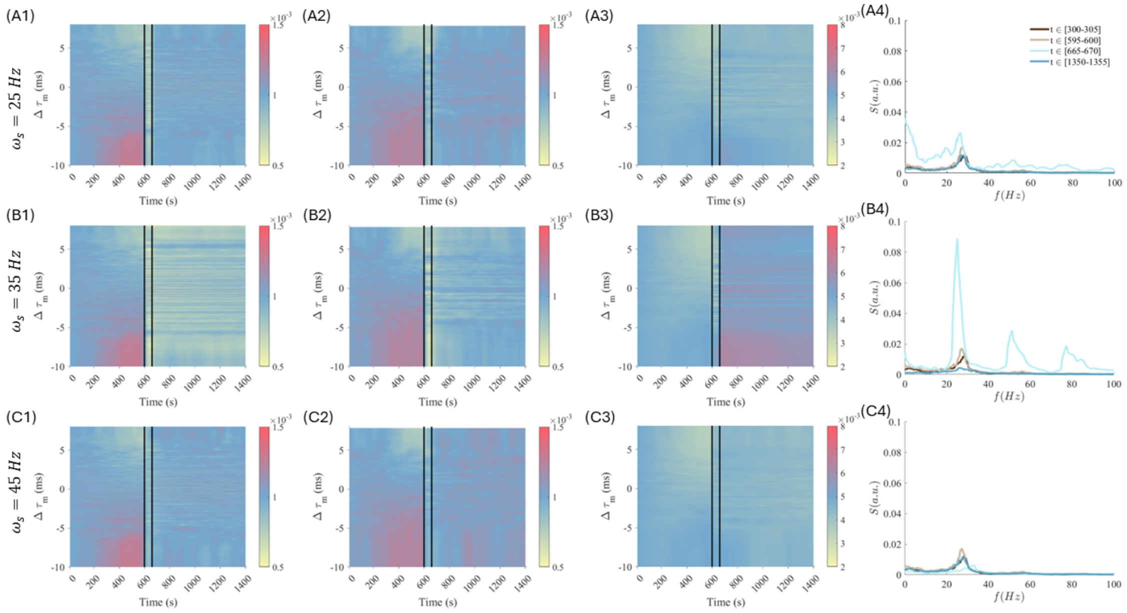

Fig S7. MTC heterogeneity shapes synaptic weights dynamics and spectral power in a frequency- and cell-type specific manner. Figure groups A, B, and C (i.e., A1-A4) are related to the stimulation frequencies $\omega_s = 25,\ 35,$ and $45\ Hz$, respectively. The heat-map plots show the dynamics of synaptic weights over time (x-axis) between synapses, which we sorted according to their MTC difference (y-axis), $\Delta\tau_m = \tau_m^{pre} - \tau_m^{post}$. Figures in each of the columns (first, second, and third columns), from left to right, depict the evolution of the synaptic weights between $E \rightarrow E$, $E \rightarrow I$, and $I \rightarrow E$, respectively, for the 1400s (simulation time). Vertical lines in each panel divided the simulation into three epochs: the pre-stimulation ($t = [0\ 600)s$), stimulation ($t = [600\ 660)s$), and post-stimulation ($t = [660\ 1400)s$) epochs. In the rightmost column, (A4), (B4), and (C4), the power spectrum of neuronal population rhythm for pre- and post-stimulation epochs is plotted. For better comparison, we preserved the same y-axis range for all panels.

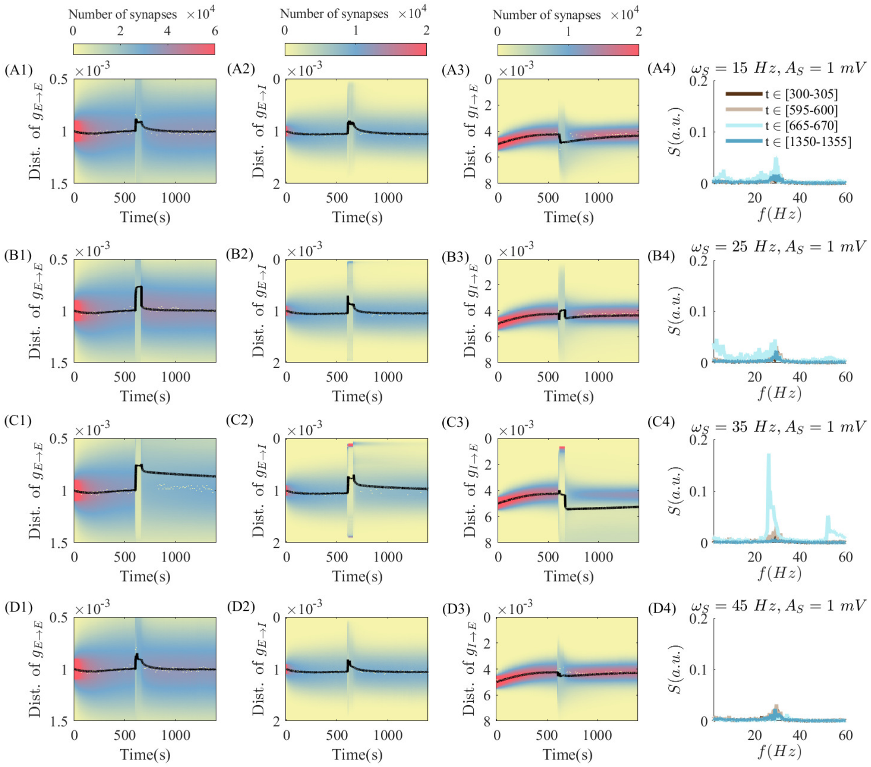

Fig S8. Time evolution of distribution of synaptic weights, steady state measure, and power spectrum of population activity. Figure groups A, B, C, and D show the synaptic weights among three sets of synaptic connections: from left to right $E \to E$, $E \to I$, and $I \to E$, respectively. The last column, (A4, B4, C4, and D4) show the power spectrum of LFP in the specified time period. The stimulation frequency, ω_s is 15, 25, 35, and 45 Hz from top row to bottom. The sum of all the synaptic weights in each aforementioned set of synaptic connections is depicted as a black line in each panel. This is, indeed, a steady-state measurement. The change in overall synaptic weights reaches a steady state after about 600 seconds. The stimulation is applied for 60 seconds (i.e. $t \in [600\ 660)s$) with an amplitude of $A_s = 1\ mV$.

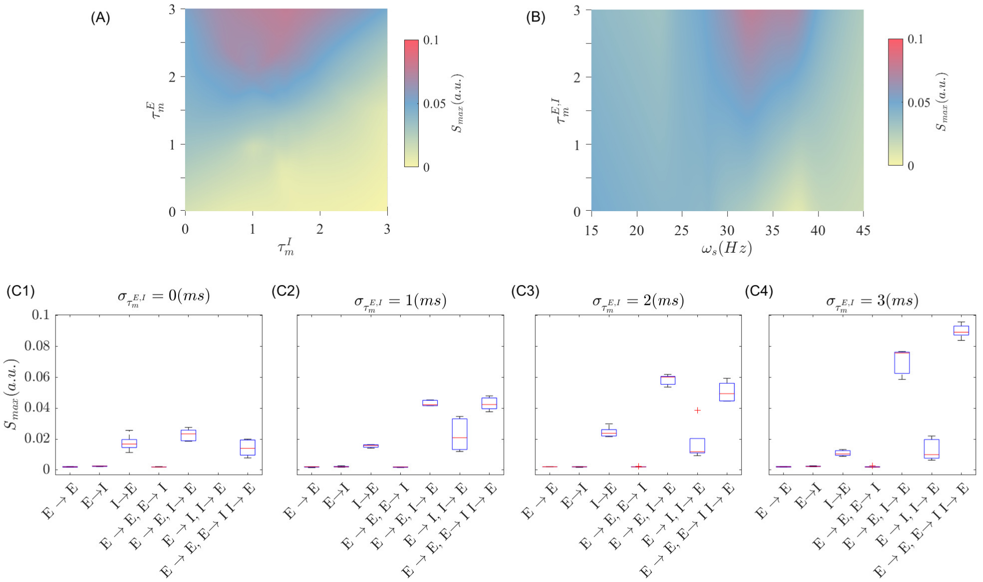

Fig S9. MTC heterogeneity amongst cell types modulates post-stimulation oscillation power. (A) Shows the peak spectral power in the post-stimulation epoch as the level of MTC heterogeneity of E (i.e., $\sigma_{\tau_m^E}$) and I (i.e., $\sigma_{\tau_m^I}$) cells is varied independently. The MTC distributions were drawn from Gaussian distribution, and $\sigma_{\tau_m^{E,I}}$ refers to the standard deviation.. (B) shows the peak spectral power in the post-stimulation epoch as a function of stimulation frequency (ω_s) and when the standard deviation (σ_{τ_m}) of MTC's distribution of both E and I cells is varied. Panels (C1) to (C4) show the changes in the peak spectral power in the post-stimulation epoch, while STDP is active only between the indicated groups of neurons along the horizontal axis, and for different values of $\sigma_{\tau_m^{E,\ I}}$, respectively. In these plots $\omega_s = 35\ Hz$ and $A_s = 1\ (mV)$.
